# Contrast-induced encephalopathy with visual and auditory hallucinations triggered by coronary angiography with iodixanol: a case report

**DOI:** 10.1186/s43044-024-00589-w

**Published:** 2024-12-02

**Authors:** Michał Kuzemczak, Sławomir Gołębiewski

**Affiliations:** 1 Department of Interventional Cardiology and Internal Diseases, Military Institute of Medicine – National Research Institute, Zegrzyńska 8 Street, 05-119 Legionowo, Poland; 2 Department of Medical Rescue, Chair of Emergency Medicine, Poznan University of Medical Sciences, Poznań, Poland; 3 Catheterization Lab, Biegański Hospital, Department of Cardiology, Medical University of Lodz, Łódź, Poland

**Keywords:** Contrast-induced encephalopathy, Contrast dye, Iodixanol, Coronary angiography

## Abstract

**Background:**

Contrast-induced encephalopathy (CIE) is a rare complication of coronary angiography posing a significant diagnostic challenge. Its incidence has substantially declined with the introduction of nonionic low-osmolar contrast media and, in most cases, it manifests with transient cortical blindness. Concomitant visual and auditory hallucinations in the course of CIE have never been reported.

**Case report:**

We present the first reported case of CIE with concomitant visual and auditory hallucinations following coronary angiography in an 80-year-old female patient. The procedure was elective and performed via right radial approach. During the procedure, significant difficulties in crossing a tortuous and calcified brachiocephalic trunk were encountered. The patient lost awareness of time and place, became agitated and started having aphasia. Periprocedural stroke was suspected as a consequence of atherosclerotic plaque mobilization and dislodging atheromatous material to the cerebral vasculature. The patient became fully oriented without aphasia within 24 h, but started having auditory and visual hallucinations. Stroke was excluded by an urgent MRI, and ultimately CIE was diagnosed. A supportive therapy with sedation and intravenous hydration was used with subsequent commencement of quetiapine treatment for hallucinations. The symptoms resolved after 5 days, and quetiapine was successfully discontinued.

**Conclusions:**

Based on the unique case report, CIE may manifest with concomitant visual and auditory hallucinations. In some instances, the clinical entity may mimic stroke; therefore, it is crucial to rule out this acute neurological condition and prevent patients from receiving a potentially harmful treatment.

**Supplementary Information:**

The online version contains supplementary material available at 10.1186/s43044-024-00589-w.

## Introduction

Since administration of iodinated contrast agents is an inherent part of interventional coronary procedures, adverse effects associated with these media should be clinically anticipated. Although their incidence has substantially declined with the introduction of nonionic low-osmolar media, they are still encountered in clinical practice and may pose a significant diagnostic challenge. One of the most rare complications is contrast-induced encephalopathy (CIE) [1]. In the systematic review published by Spina et al., only a total of 52 cases were reported in the literature between 1970 and 2017 [2]. The underlying mechanism of the clinical entity is still a matter of a debate, but the most plausible one is contrast media-related shrinkage of endothelial cells within blood–brain barrier (BBB) leading to penetration of contrast dye into a patient’s central nervous system, neuronal toxicity and cerebral edema [2, 3]. CIE may manifest with a plethora of symptoms (e.g., visual disturbances—52% of cases, focal motor and sensory deficits—28.8% of cases, seizures—17.3% of cases, global aphasia—13.5% of cases) mimicking several disorders, including debilitating stroke [1–4]. Herein, we present an extremely rare case of CIE with visual and auditory hallucinations triggered by coronary angiography with administration of iodixanol.

## Case report

An 80-year-old female patient with chronic coronary syndrome, type 2 diabetes, hypertension and dyslipidemia was admitted for a coronary angiography. She was fully oriented in time and place without any neurological signs. At the admission, the patient’s lipid profile was well-controlled (LDL 65 mg/dL, HDL 80 mg/dL, total cholesterol 155 mg/dL, triglycerides 120 mg/dL) on 20 mg rosuvastatin, while random blood glucose level on oral medications was slightly elevated (147 mg/dL). Her creatinine level was within normal range (0.8 mg/dL). Complete blood count (CBC), TSH and electrolytes were also unremarkable. Overall, the patient was stable and well-managed with no significant clinical concerns at the admission. The personal as well as family neurological and psychiatric history were unremarkable. Sixteen years earlier she had undergone a coronary procedure without any neurological abnormalities.

**Fig. 1:**
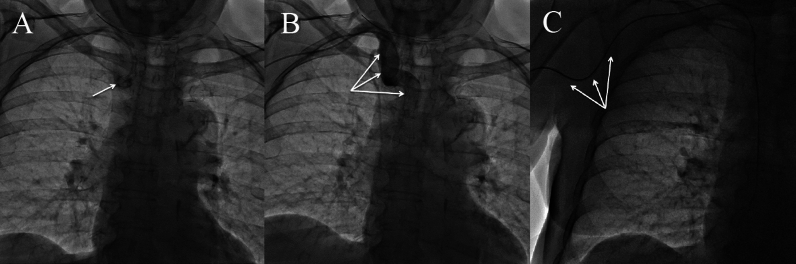
**A** Severe calcifications of the tortuous brachiocephalic trunk (white arrow); **B** angiography of the tortuous brachiocephalic trunk (white arrows); **C** a high tension applied on the catheter due to resistance met during its advancement (white arrows indicating bending of the catheter in the proximal part of the brachial artery and axillary artery)

**Fig. 2:**
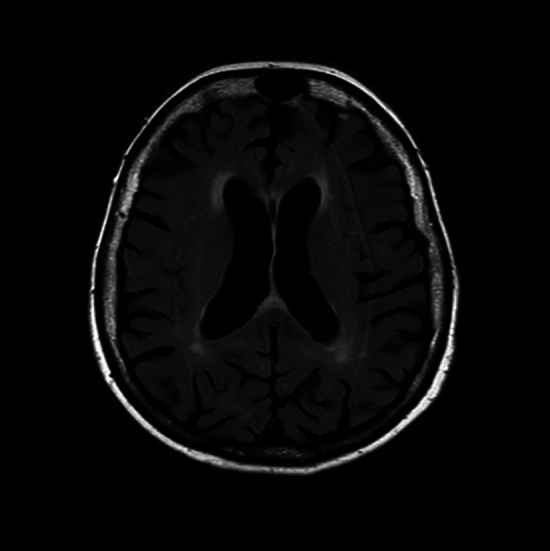
MRI demonstrating white matter hyperintensities (WMHs) with no features of stroke or intracranial bleeding

The present procedure was performed via right radial approach with the use of 120 ml of contrast (iodixanol). During the procedure, a tortuosity with pronounced calcifications was encountered in the patient’s brachiocephalic trunk (Fig. 1A, B Supplementary material—Video 1). Therefore, passing a catheter and engaging the left coronary artery ostium were challenging and required forced manipulations generating a high tension along the catheter (Fig. 1C). After the left coronary angiography and before exchanging diagnostic catheters, the patient lost awareness of time and place, became agitated and started having aphasia. Given the cumbersome vascular anatomy and obstacles during the procedure, a periprocedural stroke was suspected as a consequence of atherosclerotic plaque mobilization and dislodging atheromatous material to the cerebral vasculature. Surprisingly, a subsequent urgent MRI excluded stroke and demonstrated white matter hyperintensities (WMHs) on T2-weighted images (Fig. 2)*.* No focal neurological deficits were detected on physical examination and, within the next 24 h, she became fully oriented and alert without aphasia or impaired comprehension. Noteworthy, she started having visual (the patient’s quote “*I can see beautiful flowers and animals, mainly giraffes”*) and auditory (the patient’s quote “*I can hear weird voices”*) hallucinations with a full retention of insight (awareness of the unreal nature of hallucinations). Metabolic disorders were excluded since all the relevant laboratory tests were unremarkable. Furthermore, mental disorders were also ruled out by means of a structured psychiatric interview. Electroencephalography did not reveal epileptiform waves. Ultimately, given the clinical findings and WMHs on MRI, a contrast-induced encephalopathy (CIE) was diagnosed. A supportive therapy with sedation (benzodiazepine, i.e., diazepam 5 mg twice daily, and haloperidol 5 mg once daily for agitation) and intravenous hydration (0.9% sodium chloride intravenous infusion twice daily) was initially used for 4 days, with a subsequent commencement of quetiapine (25 mg twice daily) treatment for hallucinations. The symptoms resolved after 5 days, and quetiapine was successfully discontinued.

## Discussion

Left heart catheterization may cause cerebral microemboli leading to acute ischemic stroke with morphological changes in a brain and acute cognitive impairment [5]. However, cognitive abnormalities can be also related to the use of contrast dye and its influence on central nervous system function. The present case report describes a unique presentation of CIE with concomitant visual and auditory hallucinations. To the best of our knowledge, this is the first case of a patient with such a manifestation of CIE triggered by coronary angiography.

The underlying pathomechanism of CIE still remains a matter of debate. The most plausible one is a “disruption” of BBB integrity caused by a contrast agent and its direct neurotoxic effect [1, 4]. The overwhelming majority of CIE cases are reversible and have no significant long-term effects. Patients spontaneously recover within hours or days (as presented in this case); however, more severe and fatal cases have also been reported [6, 7]. Interestingly, the present patient started having visual and auditory hallucinations lasting for 5 days. While the formers have been reported (two cases to date), the latters have never been documented in the course of CIE [8].

CIE may present with a variety of symptoms (confusion, headache, seizures and rarely focal neurological deficits), but the most common manifestation is transient cortical blindness, presumably due to higher occipital BBB permeability [1, 4]. We posit that this mechanism was responsible for visual hallucinations in the presented case. The auditory hallucinations are a completely novel finding requiring further investigation.

From clinical standpoint, the key issue is to identify patients at risk, and to establish preventive and therapeutic strategies. Hypertension, diabetes, renal impairment, impaired cerebral autoregulation and male gender are among risk factors for CIE [1]. A type of a contrast agent is also of vital importance. Iodixanol which was used in the present case is a nonionic iso-osmolar agent, less neurotoxic compared to older hypertonic contrast media. However, even a low volume of this agent may potentially cause a devastating CIE [7]. Interestingly, post-CIE patients do not necessarily develop this complication again upon reexposure to contrast dye [9]. The above-mentioned findings imply that CIE occurs in a sporadic fashion at any dosage, and with all types of contrast agents. As a consequence, the unpredictability of CIE recurrence may prevent health professionals from referring post-CIE patients for coronary procedures, even when they are strongly indicated, which might be one of the underestimated and detrimental effects of CIE.

Currently, no specific preventive measure or treatment is available. The use of the lowest possible volume of less neurotoxic contrast agents is recommended. Steroids and mannitol are anecdotally used, but no reliable data on this have been provided to date [1, 6]. Finally, and perhaps most importantly, given the self-limiting nature of CIE in vast majority of cases, it is crucial to exclude more deleterious clinical conditions and prevent a patient from receiving a potentially harmful treatment (e.g., thrombolytic therapy).

## Conclusions

The present case report shows that CIE may manifest with concomitant visual and auditory hallucinations. In this particular patient, the symptoms were reversible with no long-term clinical sequela. Despite several unanswered questions deserving further attention, one can expect that studies providing mechanistic insights into CIE and yielding conclusions relevant to day-to-day practice will likely be hampered by the rarity of the clinical entity.

## Supplementary Information

Additional file1: Supplementary material 1: Video 1: Angiography of the tortuous brachocephalic trunk.

## Data Availability

All relevant data have been included in the article.

## Abbreviations

BBB: Blood–brain barrier
CIE: Contrast-induced encephalopathy
MRI: Magnetic resonance imaging
WMHs: White matter hyperintensities
